# Advances in the Synthesis of Ring-Fused Benzimidazoles and Imidazobenzimidazoles

**DOI:** 10.3390/molecules26092684

**Published:** 2021-05-04

**Authors:** Martin Sweeney, Darren Conboy, Styliana I. Mirallai, Fawaz Aldabbagh

**Affiliations:** 1 School of Chemistry, National University of Ireland Galway, University Road, H91 TK33 Galway, Ireland; M.Sweeney5@nuigalway.ie (M.S.); styliana.mirallai@nuigalway.ie (S.I.M.); 2 Department of Pharmacy, School of Life Sciences, Pharmacy and Chemistry, Kingston University, Penrhyn Road, Kingston upon Thames KT1 2EE, UK; D.Conboy@kingston.ac.uk

**Keywords:** green chemistry, halogen, heterocycle, hydrogen peroxide, imidazole, iodine, nitrosobenzene, oxone, palladium, quinone

## Abstract

This review article provides a perspective on the synthesis of alicyclic and heterocyclic ring-fused benzimidazoles, imidazo[4,5-*f*]benzimidazoles, and imidazo[5,4-*f*]benzimidazoles. These heterocycles have a plethora of biological activities with the iminoquinone and quinone derivatives displaying potent bioreductive antitumor activity. Synthesis is categorized according to the cyclization reaction and mechanisms are detailed. Nitrobenzene reduction, cyclization of aryl amidines, lactams and isothiocyanates are described. Protocols include condensation, cross-dehydrogenative coupling with transition metal catalysis, annulation onto benzimidazole, often using CuI-catalysis, and radical cyclization with homolytic aromatic substitution. Many oxidative transformations are under metal-free conditions, including using thermal, photochemical, and electrochemical methods. Syntheses of diazole analogues of mitomycin C derivatives are described. Traditional oxidations of *o*-(cycloamino)anilines using peroxides in acid via the *t*-amino effect remain popular.

## 1. Introduction

### 1.1. Significance and Biological Activity

**Figure 1:**
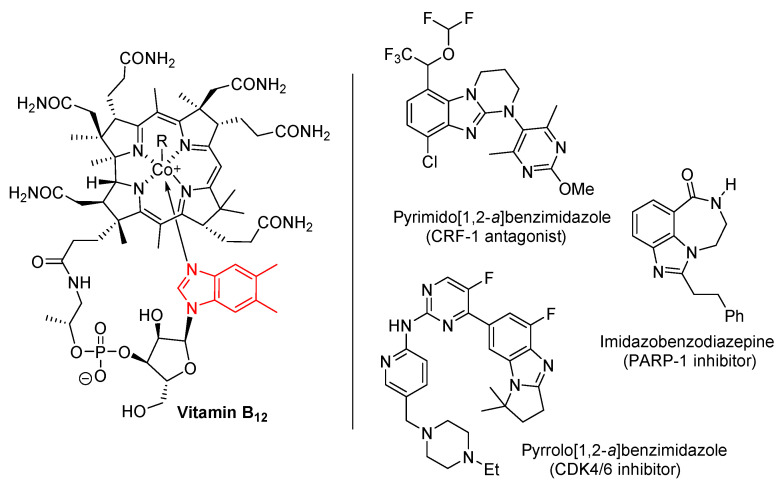
Benzimidazole in vitamin B_12_ and biologically-active ring-fused systems [1,2,3,4,5,6,7,8,9].

Benzimidazole is an important heterocyclic pharmacophore. Therapeutic interest was aroused in the 1950s, when 5,6-dimethylbenzimidazole was discovered as a degradation product of vitamin-B_12_ (Figure 1) [1,2]. Benzimidazoles possess a wide range of biological activities [3,4,5,6], significantly utilized as APIs in medicines [5], and as pesticides [6]. Moreover among the ring-fused benzimidazoles, pyrimido[1,2-*a*]benzimidazoles were discovered as effective corticotropin releasing factor-1 (CRF-1) receptor antagonists for potential treatment of mental health disorders [7]. A pyrrolo[1,2-*a*]benzimidazole was found to be the most effective cyclin-dependent kinase 4/6 (CDK4/6) inhibitor evaluated within a series of highly functionalized five to seven-membered alicyclic ring-fused benzimidazoles [8]. Other ring-fused benzimidazoles, the imidazobenzodiazepines were investigated as poly(ADP-ribose) polymerase (PARP-1) inhibitors, allowing reduction of hyperglycemia with neuroprotective effects in animal models [9]. 

**Figure 2:**
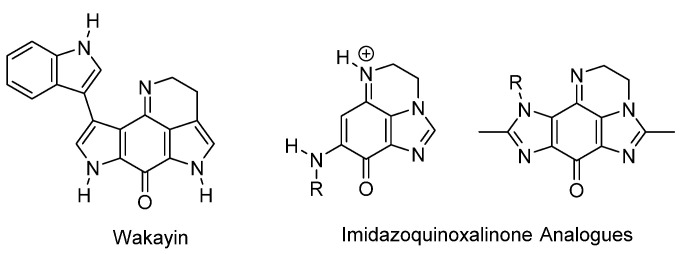
Imidazole-based wakayin analogues [10].

**Figure 3:**
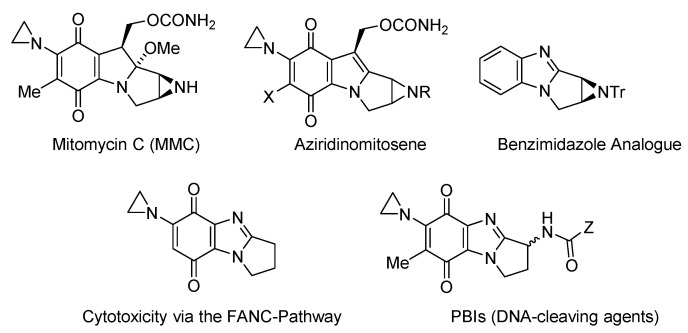
Aziridinyl-functionalized antitumor agents [11,12,13,14,15,16,17,18].

**Figure 4:**
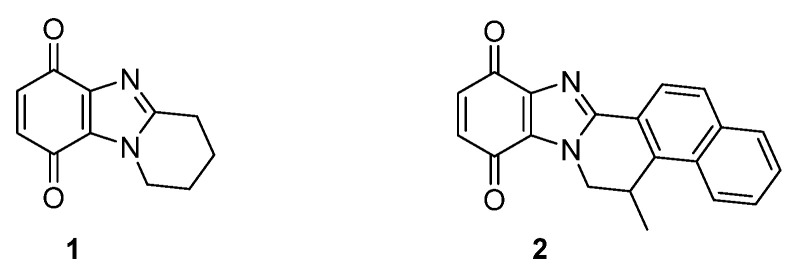
Benzimidazolequinones targeting over-expressed reductases in solid tumors [21,22,23].

**Figure 5:**
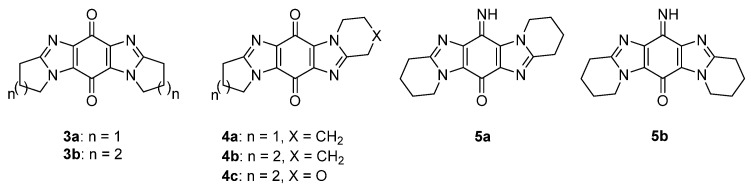
Imidazobenzimidazolequinones and iminoquinones [24,25,26,27,28,29].

The most widely studied of the applications of ring-fused benzimidazoles and imidazobenzimidazoles is as bioreductive antitumor agents, when the fused benzene part is an iminoquinone or quinone functionality (Figure 2, Figure 3, Figure 4 and Figure 5). Indole-based natural products often inspire analogue synthesis, including imidazoquinoxalinone analogues of the marine natural product, wakayin [10], a topoisomerase I inhibitor (Figure 2). 

Mitomycin C (MMC) has, however, attracted most interest, as the archetypal bioreductive antitumor antibiotic, which cross-links to DNA [11]. Incorporation of the DNA-alkylating aziridinyl moiety leads to cytotoxicity via the FANC DNA-repair pathway [12,13], and the benzimidazole analogue of the bioactivated form of MMC (aziridinomitosene) was prepared and evaluated against breast cancer cell lines [14] (Figure 3). Skibo has published extensively on aziridinylpyrrolo[1,2-*a*]benzimidazolequinones (PBIs) as DNA cleaving agents with analogues showing melanoma-specific cytotoxicity [15,16,17,18].

One or two-electron reductases are responsible for bioreductive activation. NADPH-cytochrome *c* (P450) reductase is predominant under hypoxic conditions with the one-electron reduction reversed by oxygen [19]. Many solid tumors also over-express the obligatory two-electron reductase NAD(P)H:quinone oxidoreductase 1 (NQO1, also known as DT-diaphorase), which is a popular target for anti-cancer studies [20]. Many anti-cancer agents do not contain conventional DNA damaging functionality, and cytotoxicity may be due to the formation of reactive oxygen species. Pyrido[1,2-*a*]benzimidazolequinone **1** (Figure 4) is more than 300 times more cytotoxic under hypoxic conditions than the clinical drug, MMC (Figure 3), with cytotoxicity for alicyclic ring-fused benzimidazoles correlated to reductive potentials [21,22]. Highly conjugated naphthyl fused benzimidazolequinone **2** leads to increased stability of reduced intermediates leading to specificity towards human cancer cell lines over-expressing NQO1 [23]. 

Ring-fused imidazo[4,5-*f*]benzimidazolequinones **3a** and **3b** are NQO1 substrates [24,25], with **3a**, at the National Cancer Institute (NCI), showing specificity towards the killing of melanoma cell lines (Figure 5) [24]. Our group was the first to provide viable synthetic protocols for accessing ring-fused imidazo[5,4-*f*]benzimidazoles, enabling evaluation of quinone and iminoquinone derivatives for toxicity against cancer cell lines [26,27,28,29]. Compared to alicyclic ring-fused analogues **4a** and **4b**, the oxygen atom of the 1,4-oxazino ring was found to increase toxicity of **4c** [27]. Iminoquinone **5a** isolated from the Fremy oxidation to prepare **4b**, was unexpectedly the most potent imidazobenzimidazole, with more than 12 times greater cytotoxicity towards a prostate cancer cell line (DU145) than a normal fibroblast cell line (GM00637) [26]. More intensive cytotoxicity assays, computational docking, and NCI COMPARE analysis on **5a**, revealed good correlation with NQO1 [28]. In contrast, isomeric imidazo[4,5-*f*]benzimidazole **5b** was inactive against the NCI 60 cell line panel [29]. 

### 1.2. Available Synthetic Methods

**Scheme 1:**
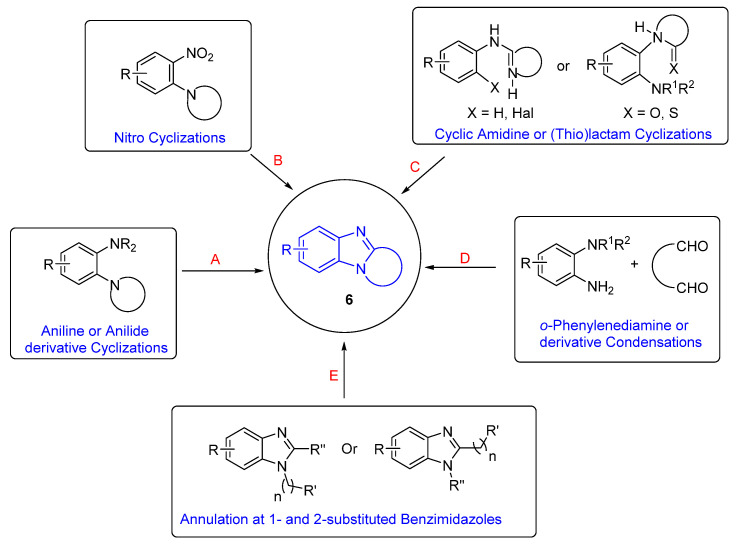
Categorizing available synthetic methods according to the cyclization reaction A-E.

The categories of syntheses of ring-fused benzimidazoles **6** are according to the cyclization reaction (Scheme 1). Oxidative cyclizations from aniline or anilide derivatives is the most studied route (Route A) and is presented in context with the plethora of other syntheses that build the benzimidazole moiety (Routes B–D). The section on Route A is sub-divided into syntheses of benzimidazole and imidazobenzimidazole scaffolds. Lastly, there is a section on syntheses, which begin with the benzimidazole moiety (Route E), sub-divided according to reaction (type) conditions. This is not an exhaustive review, and the reader should consult reviews on polycyclic benzimidazoles for comprehensive lists of syntheses [30,31,32,33]. Since the late 1990s, Aldabbagh et al. have worked on the discovery of new ring-fused benzimidazoles and synthetic methods, and the collated articles related to their research are reviewed herein. A reviewer recommended a Scifinder^n^ search of “benzimidazole-fused”, which was completed, and significant references are incorporated. For brevity, full papers are cited and not the preceding communication article. A historical perceptive is taken and analysis of the most significant contributions to the field is carried out. In particular, methodology that forms a variety of ring-fused benzimidazoles is of interest, rather than procedures that give mainly the benzimidazole core. 

## 2. Syntheses of Ring-Fused Benzimidazoles and Imidazobenzimidazoles

### 2.1. Oxidations of o-Cycloaminoanilines and Anilide Derivatives (Route A)

There are distinct differences in the reaction mechanisms and conditions for ring-fused benzimidazole and imidazobenzimidazole formation warranting sub-division. Benzimidazoles form by oxidative cyclization of anilines via nitrosobenzene intermediates; in contrast, cyclization to give the ring-fused imidazobenzimidazole must begin from anilides and proceed via amine *N*-oxide intermediates under acidic conditions. 

#### 2.1.1. Forming Ring-Fused Benzimidazoles

**Scheme 2:**
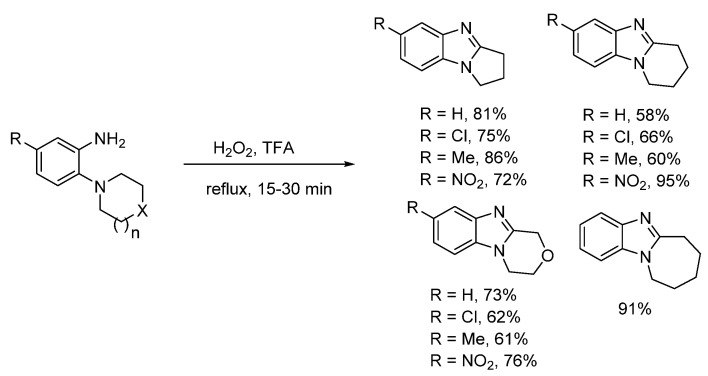
Nair and Adams oxidative cyclizations of anilines [36].

**Scheme 3:**
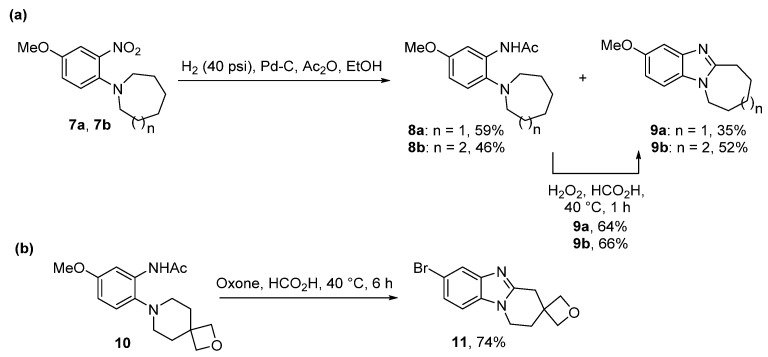
Acetanilide cyclizations onto (**a**) azepino- and azocino- [39], and (**b**) spirocyclic oxetane- [40] derivatives.

In 1908, Spiegel and Kaufmann reported that Caro’s acid (peroxymonosulfuric acid, H_2_SO_5_) oxidized 5-nitro-2-(piperidin-1-yl)aniline to 7-nitro-1,2,3,4-tetrahydropyrido[1,2-*a*]benzimidazole [34]. In the absence of the nitro-substituent, no oxidative cyclization occurred. Caro’s acid was already known to oxidize anilines to nitrosobenzenes [35], so supporting the idea of a nitroso intermediate. The prominent 20th century chemist and creator of Adam’s catalyst, Roger Adams with Nair refined this methodology, and accessed a range of five to seven-membered ring-fused benzimidazoles in good to high yields using peroxytrifluoroacetic acid generated in situ from H_2_O_2_ and trifluoroacetic acid (TFA) (Scheme 2) [36]. Six-membered cyclization yields were higher, when the anilines contained a nitro-substituent. Meth-Cohn and Suschitzky [37] soon refuted the observation made by Nair and Adams that acyl derivatives do not undergo cyclization to give benzimidazoles. These workers showed a range of anilide derivatives (formyl, acetyl and benzoyl) underwent oxidative cyclizations using peroxytrifluoroacetic acid or performic acid (H_2_O_2_ and HCO_2_H). Meth-Cohn preferred the use of *o*-cyclic amine substituted anilides as substrates for making ring-fused benzimidazoles [37,38]. Meth-Cohn commented that Nair and Adams [36] had possibly formed the anilide in situ, due to the initial addition of TFA followed by H_2_O_2_ [38]. Mechanisms were proposed for benzimidazole formation from anilide, via an amine-*N*-oxide rather than the nitroso intermediate (see Section 2.1.2). Later the use of anilide derivatives as substrates for preparation of ring-fused benzimidazoles would become commonplace (Scheme 3) [13,15,27,37,38,39,40]. 

Significant amounts of seven- and eight-membered ring-fused [1,2-*a*]benzimidazoles **9a** and **9b** were formed from nitrobenzenes **7a** and **7b** during the one-pot catalytic hydrogenation and acetylation to give acetamides **8a** and **8b** (Scheme 3a) [39]. It seemed that the conformation of these large alicyclic rings favored advantageous cyclization of nitroso intermediates formed during the hydrogenation process. Acetamides **8a** and **8b** were cyclized using performic acid to **9a** and **9b** in good yields, with the former transformed to 3-aziridinylazepino[1,2-*a*]benzimidazolequinone [13,39]. A readily available and safer alternative to Caro’s acid is Oxone (2KHSO_5_·KHSO_4_·K_2_SO_4_) [41]. Due to the absence of organic waste products, Oxone in the presence of formic acid gives ring-fused benzimidazole [27,40] and imidazobenzimidazole [27] adducts without the requirement for chromatography. 2-Oxa-7-azaspiro[3.5]nonane acetamide **10** gave the spirocyclic oxetane ring-fused benzimidazole **11** in good yield by simple organic extraction from the basified aqueous mixture (Scheme 3b) [40]. 

**Scheme 4:**
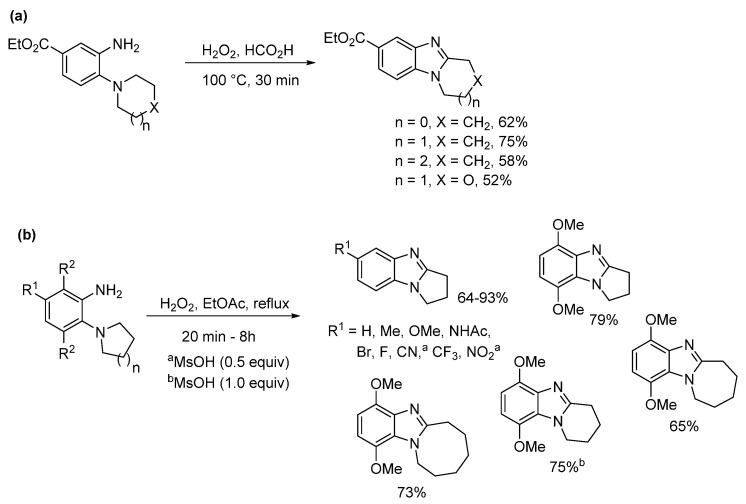
Aniline cyclizations using (**a**) performic acid [42] and (**b**) acid-free conditions [43].

Preparations of ring-fused benzimidazoles using *o*-cyclic amine substituted anilines with performic acid compare favorably with the derivative anilide reaction, with Smalley et al. reporting moderate to good yields for five- to seven-membered adducts (Scheme 4a) [42]. In a more recent study, recyclable ethyl acetate (EtOAc) replaced formic acid, with aqueous effluent, organic-aqueous extraction and chromatography avoided for the preparation of pyrrolo[1,2-*a*]benzimidazoles from commercial *o*-(pyrrolidin-1-yl)anilines (Scheme 4b) [43]. Although, the presence of strong electron-withdrawing substituents (NO_2_, CN) and the six-membered cyclization required methanesulfonic acid (MsOH) to reach high yields. However, MsOH is a green acid undergoing biodegradation by forming CO_2_ and sulfate [44].

**Scheme 5:**
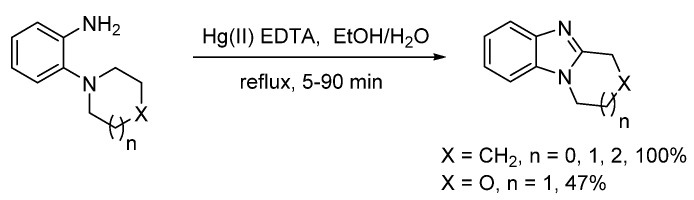
Hg(II)-mediated oxidative synthesis [46].

Alternatives to peroxide-based oxidizing systems, include MnO_2_ in cold chloroform, but yields of ring-fused benzimidazoles from *o*-cycloaminoanilines were 15–20% due to presumed formation of azo-compounds [45]. Möhrle and Gerloff reported the use of a Hg(II) EDTA complex to deliver ring-fused benzimidazoles, in quantitative yield, apart from the morpholino compound, synthesized in 47% yield (Scheme 5) [46].

**Scheme 6:**
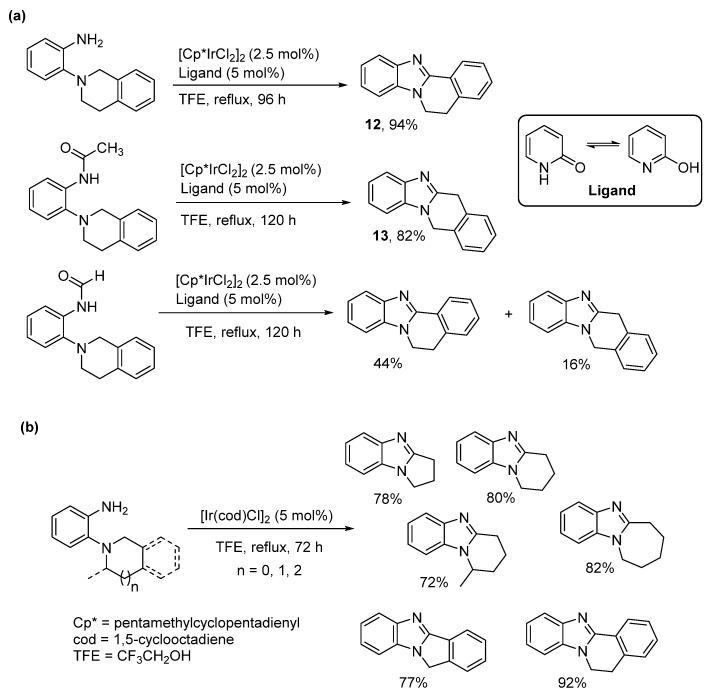
Ir(III)-mediated cyclizations (**a**) with ligand [47] and (**b**) without ligand [48].

The cross dehydrogenative coupling (CDC) involves forming the C-N bond directly from C-H and N-H bonds under oxidative conditions with a formal loss of H_2_, in a process often catalyzed by transition metals. CDC is used to describe pentamethylcyclopentadienyl Ir(III)dichloride ([Cp*IrCl_2_]_2_) catalyzed oxidative cyclization of *o*-tetrahydroisoquinoline substituted aniline derivatives (Scheme 6a) [47]. The bulk around the primary amine dictated regioselectivity. The *o*-cyclic amine substituted aniline gave the benzimidazo[2,1-*a*]isoquinoline **12**, while the more hindered acetamide derivative gave the alternative kinetic product **13**. The formamide has less steric bulk than the acetamide forming a mixture of the thermodynamic and kinetic products. The reaction was extended to the synthesis of pyrrolo-, pyrido-, and azepino-[1,2-*a*]benzimidazoles, without the requirement for a ligand (Scheme 6b), but was less successful for making morpholino- and piperazino-ring-fused analogues [48].

**Scheme 7:**
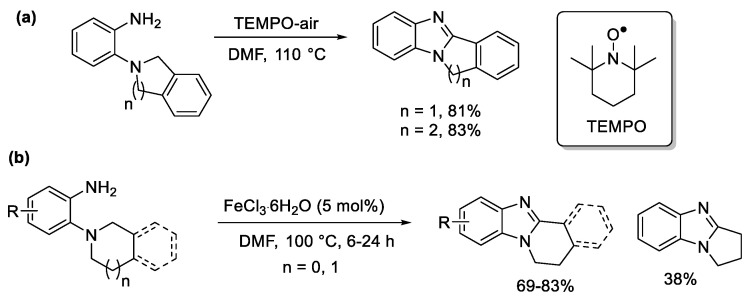
Thermal oxidative cyclizations mediated by (**a**) TEMPO/air [49] and (**b**) Fe(III) [50].

The isoindoline and tetrahydroisoquinoline substrates are the easiest to oxidize at high temperatures, including in the presence of TEMPO in air (Scheme 7a) [49], and catalytic iron(III) [50]. The latter gave the highest yields for the benzimidazo[2,1-*a*]isoquinoline systems (Scheme 7b).

**Scheme 8:**
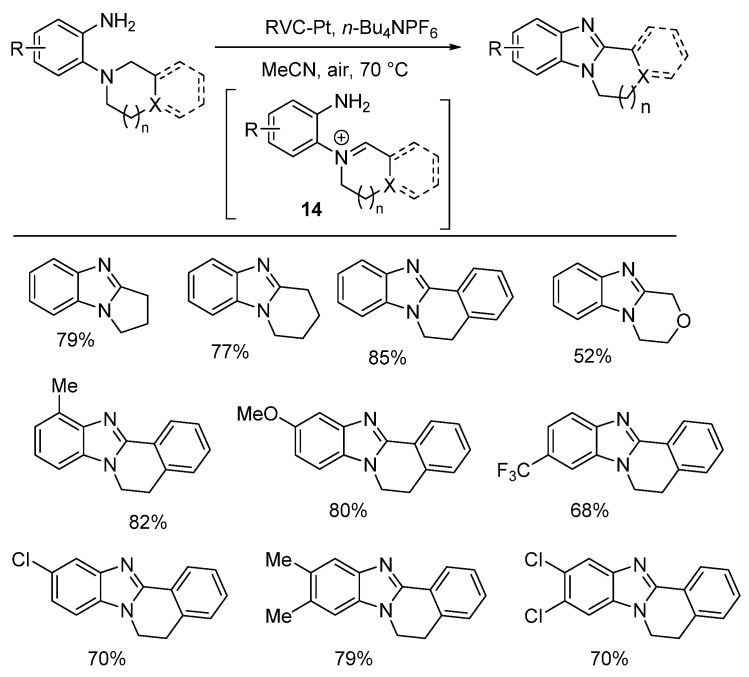
Electrochemical oxidative cyclizations [51].

Aniodic oxidation gave the required iminium ion **14** for cyclization (Scheme 8) [51]. The electrolyte was *n*-Bu_4_NPF_6_ (20 mol%), and the anode is reticulated vitreous carbon (RVC), and Pt is the cathode in an undivided cell at a constant current of 10 mA. A Russian team reported the electrochemical oxidative cyclization with reduction of nitrobenzene for cyclization onto an *o*-piperidinyl-substituent to give pyrido[1,2-*a*]benzimidazoles [52]. 

**Scheme 9:**
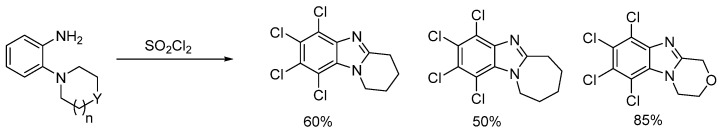
Synthesis of polychlorinated ring-fused benzimidazoles using SO_2_Cl_2_ [53].

In the early 1970s, *o*-cyclic amine substituted anilines reacted in neat sulfuryl chloride in an oxidative cyclization with concomitant tetrachlorination of the activated aromatic nucleus (Scheme 9) [53]. The attempt to tetrachlorinate the *o*-pyrrolo substituted aniline analogue led to an inseparable mixture of mono-, di-, and trichloro ring-fused benzimidazoles. 

**Scheme 10:**
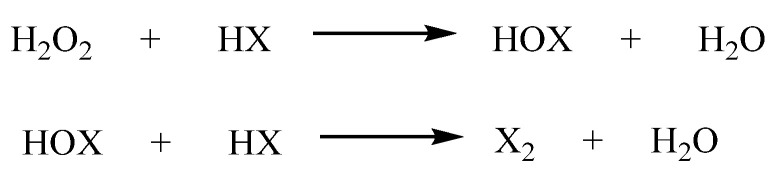
Molecular halogen (X_2_) generated from H_2_O_2_ and HX [54,55,56].

More recently, we heralded the use of H_2_O_2_ with hydrohalic acid (HX), as a convenient oxidizing and halogenation system for organic synthesis [54,55,56]. The system allows in situ generation of Cl_2_ and Br_2_, with the only by-product, being water (Scheme 10). The salt of hypochlorous acid (HOCl) is the active ingredient in domestic bleach and is the intermediate of the reaction between H_2_O_2_ and HCl. 

**Scheme 11:**
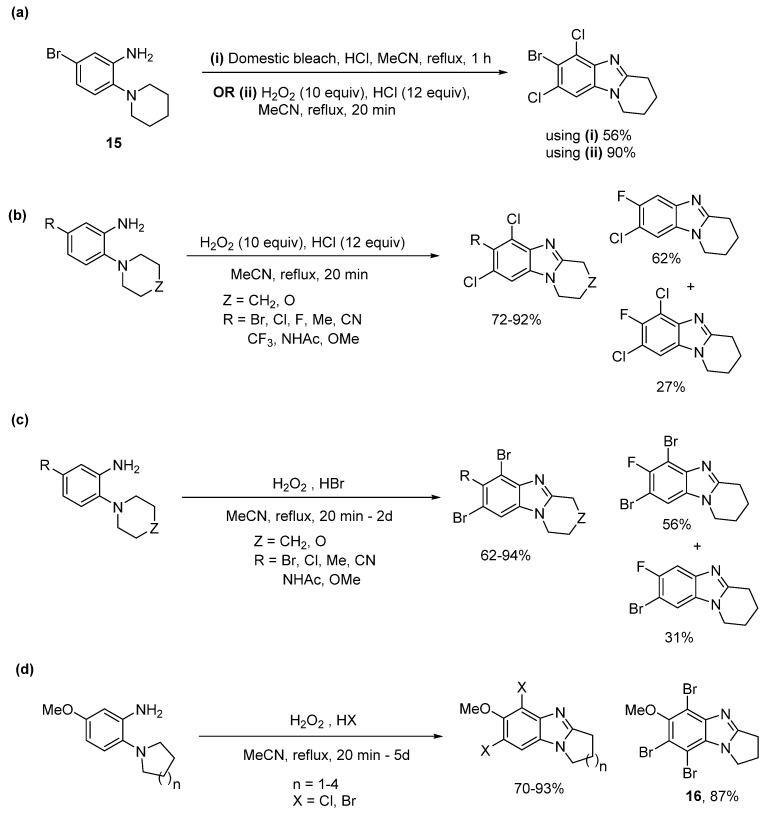
One-pot ring closure with selective halogenation using (**a**) domestic bleach or H_2_O_2_/HCl, (**b**) H_2_O_2_/HCl, and (**c**) H_2_O_2_/HBr, and (**d**) five to eight-membered ring-fused adducts [54].

Domestic bleach gave cyclization and dichlorination of aniline **15** in 56% yield (Scheme 11a), with the lower yield attributed to the requirement for chromatography to separate the additives in the bleach [54]. Moreover, using H_2_O_2_/HX a library of selectively dichlorinated and dibrominated ring-fused benzimidazoles was prepared in high yields from commercial *o*-cyclic amine substituted anilines, with most cases not requiring chromatography (Scheme 11b,c). 5-Fluoro-2-piperidinylaniline was an exception, giving significant amounts of cyclization with monochlorination or monobromination. Bromination tended to be slower than chlorination, and tribrominated product **16** was isolated for the *o*-(pyrrolidin-1-yl)aniline, due to difficulties in cleanly isolating 5,7-dibromopyrrolo[1,2-*a*]benzimidazole (Scheme 11d).

**Scheme 12:**
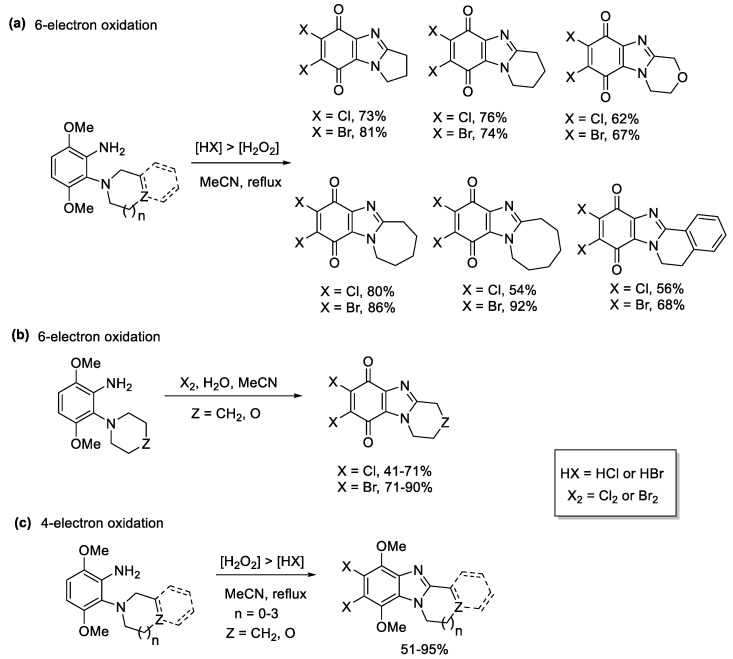
One-pot transformations of anilines to halogenated ring-fused benzimidazolequinones using (**a**) H_2_O_2_/HX, (**b**) Cl_2_ and Br_2_ with water, and (**c**) H_2_O_2_/HX without quinone formation [55].

3,6-Dimethoxy-2-(cycloamino)anilines underwent 6-electron oxidations to afford a variety of ring-fused halogenated benzimidazolequinones, when using higher amounts of HCl or HBr relative to H_2_O_2_ (Scheme 12a) [55]. The active species is the elemental halogen (X_2_) with water required for quinone formation (Scheme 12b). When less in situ halogen was generated, using [H_2_O_2_] > [HX], the 4-electron oxidation occurred, to give ring-fused halogenated benzimidazoles (Scheme 12c). 

**Scheme 13:**
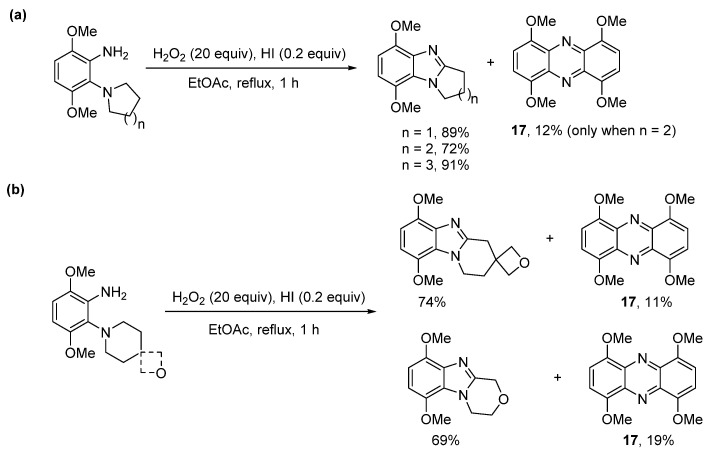
H_2_O_2_/HI-mediated (**a**) five- to seven-membered cyclizations and (**b**) six-membered cyclizations with phenazine formation [56].

**Scheme 14:**
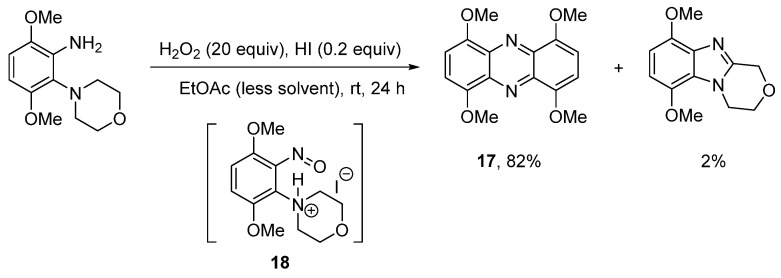
Optimizing the synthesis of 1,4,6,9-tetramethoxyphenazine **17** [56].

The use of hydroiodic acid (HI) is preferred when oxidative cyclization is required without halogenation [56], due to the relatively smaller electrophilicity of iodine [57]. Five- and seven-membered cyclizations of 3,6-dimethoxy-2-(cycloamino)anilines with H_2_O_2_ and a catalytic amount of HI in EtOAc proceeded in high yield (Scheme 13a), but 1,4,6,9-tetramethoxyphenazine **17**, was unexpectedly formed, as an orange precipitate from six-membered cyclizations (Scheme 13a,b) [56]. The absence of phenazine **17** from the five- and seven-membered cyclizations was consistent with previous observations that six-membered oxidative cyclizations are more difficult [36,43]. The formation of **17** was optimized by reducing the amount of EtOAc (the reaction solvent) by four-fold and by decreasing the reaction temperature to room temperature (Scheme 14). Moreover, the isolation of **17** was indicative of a nitrosobenzene intermediate in the conversion of *o*-(cycloamino)anilines to ring-fused benzimidazoles via the so-called *t*-amino effect [58]. Syntheses of phenazines involve nitroso intermediates [56,59,60]. Recent evidence in the synthesis of ring-fused benzimidazoles, included GC-MS of the reaction mixture (Scheme 14), after 1 h, which revealed EI-MS fragmentation pattern consistent with intermediate **18** [56].

**Scheme 15:**
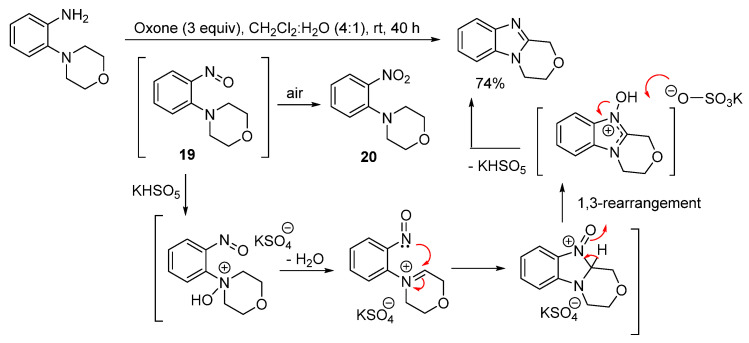
Oxone-mediated ring-closure of 2-(morpholin-4-yl)aniline [61].

3,4-Dihydro-1*H*-[1,4]oxazino[4,3-*a*]benzimidazole can however be prepared in good yield in the absence of acid from 2-(morpholin-4-yl)aniline using Oxone at room temperature (Scheme 15) [61]. Sampling the reaction at short reaction times (within 2 min), gave 4-(2-nitrophenyl)morpholine (**20**), which formed through advantageous air oxidation of the nitroso intermediate **19**. The proposed mechanism postulates that KHSO_5_ (the active ingredient of Oxone) is catalytic, consistent with the use of catalytic amounts of HI in the H_2_O_2_/HI-mediated oxidative cyclization of 3,6-dimethoxy-2-(cycloamino)anilines (Scheme 13 and Scheme 14) [56].

**Scheme 16:**
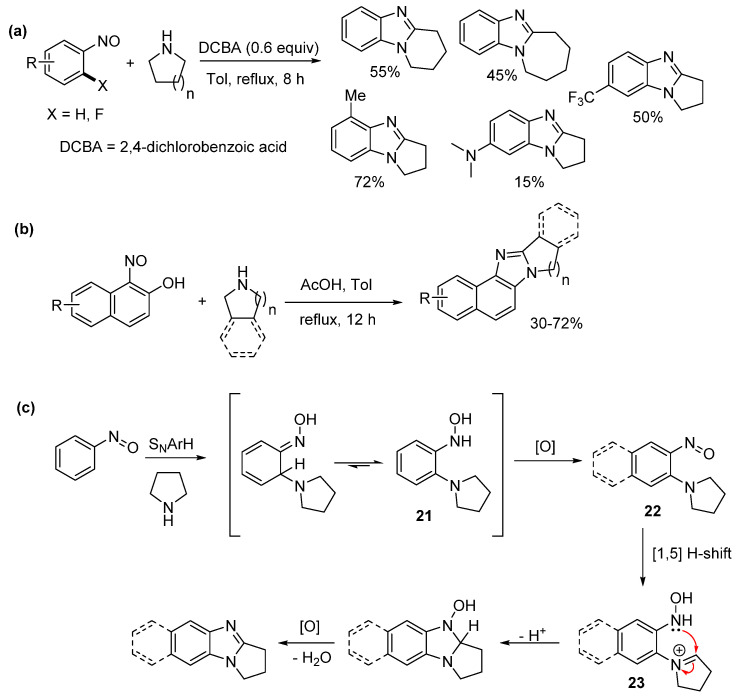
Sequential aminations of nitrosoarenes to prepare (**a**) alicyclic ring-fused benzimidazoles, (**b**) naphthoimidazoles, and (**c**) the proposed mechanism [62].

Jana et al. reported one-pot sequential amination of nitrosoarenes with alicyclic amines, followed by oxidative cyclization to give ring-fused benzimidazoles (Scheme 16a–c) [62]. Oxone oxidation of commercial anilines gave the nitrosoarene substrates. Nucleophilic aromatic hydrogen substitution (S_N_ArH) led to the pyrrolo[1,2-*a*]benzimidazoles, however piperido- and azepino-analogues required nucleophilic aromatic substitution (S_N_Ar) of 2-fluoronitrosobenzene to achieve reasonable yields for the [1,2-*a*] ring-fused benzimidazoles (Scheme 16a). Nucleophilic *ipso*-substitution of nitrosonaphthols with cyclic amines and subsequent oxidative cyclization delivered a diverse range of ring-fused naphthoimidazoles (Scheme 16b). The authors proposed a mechanism with mass spectrometry detecting the *o*-cyclic amine substituted phenyl hydroxylamine **21**, which oxidized to the *o*-cyclic amine substituted nitrosoarene **22** (Scheme 16c). A formal 1,5-hydride shift gave the key iminium ion intermediate **23** for oxidative cyclization [58]. In contrast to our recent work [43,56,61], the nitrosoarene cyclizations, were carried out using elevated temperatures and under acidic conditions [62]. 

#### 2.1.2. Forming Ring-Fused Imidazobenzimidazoles

**Scheme 17:**
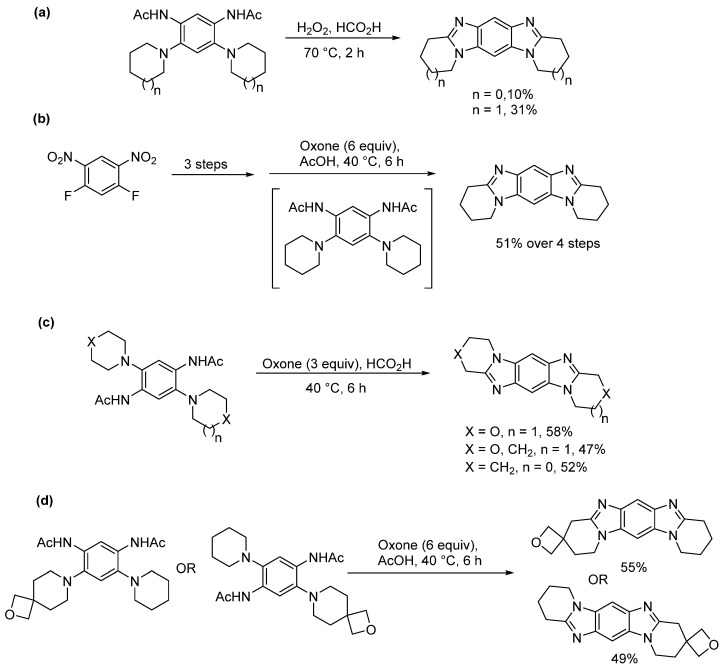
Oxidative syntheses of imidazo[4,5-*f*]benzimidazoles using (**a**) performic acid [24,25] and (**b**) Oxone [29], and (**c**) of imidazo[5,4-*f*]benzimidazoles using Oxone [27], and (**d**) with fused spirocyclic oxetane ring [61].

Oxidative cyclizations in this class date back to the 1950s and 1960s [37,63], and Skibo et al. used the performic acid mediated protocol to convert diacetamides to dipyrrolo- and dipyrido-ring-fused imidazo[4,5-*f*]benzimidazoles, but yields were low (Scheme 17a) [24,25]. One-pot double alkyl radical cyclizations onto the 2- and 6-positions of imidazobenzimidazole gave dipyrrolo-, dipyrido-, and diazepino-ring-fused imidazo[4,5-*f*]benzimidazoles and imidazo[5,4-*f*]benzimidazoles in 47–90% yield (see Section 2.5.3) [26]. However, the radical cyclization route cannot easily give imidazobenzimidazoles containing two different fused rings. Oxone in acid enabled the synthesis of symmetrical and unsymmetrical ring-fused imidazo[4,5-*f*]benzimidazoles and imidazo[5,4-*f*]benzimidazoles (Scheme 17b–d) [27,29,61]. Yields were higher than older oxidative methods [24,25,37,63], perhaps due to the easier work up, allowing isolation of the imidazobenzimidazole directly from the acidic reaction mixture using precipitation with solid Na_2_CO_3_ [27]. It is noteworthy that 49 and 55% yield for the spirocyclic oxetane ring-fused isomers, represent ≥70% yield for each ring closure [61].

**Figure 6:**
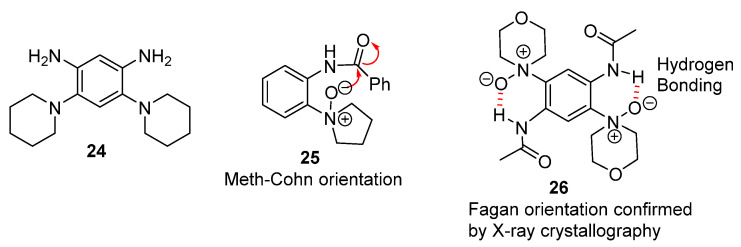
Key chemical structures in resolving the mechanism [27,37,38].

Anilide reactant and acidic conditions are a prerequisite for oxidation to the imidazobenzimidazole, with attempts to cyclize 4,6-di(piperidin-1-yl)-1,3-phenylenediamine (**24**) giving an intractable mixture (Figure 6) [61]. Meth-Cohn proposed the oxidative cyclization of acetamides to benzimidazole derivatives occurs via the amine-*N*-oxide intermediate **25** [38]. Isolated amine-*N*-oxides undergo acid-mediated benzimidazole and imidazobenzimidazole formation [27,61]. 

**Scheme 18:**
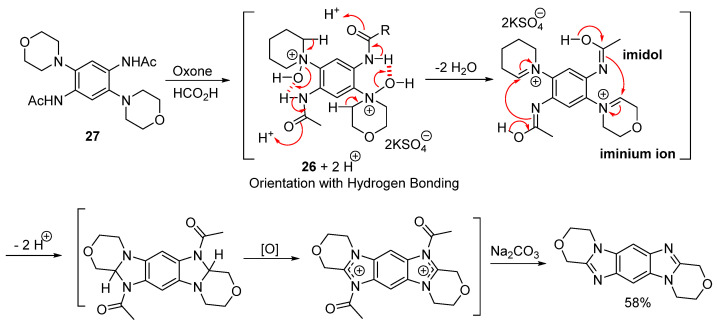
Mechanism for oxidation *o*-(cycloamino)acetamide to imidazobenzimidazole [27].

The diamine-*N*-oxide intermediate **26** for imidazo[5,4-*f*]benzimidazole formation was isolated by Fagan [27]. The X-ray crystal structure of **26** showed hydrogen bonding between the amide NH and the amine *N*-oxide residues, supporting the absence of the amide NH peaks in the ^1^H NMR spectra of solutions of amine *N*-oxides [27,38,61]. This is contrary to the orientation of the amine-*N*-oxide **25** adopted in Meth-Cohn’s Polonovski-type reaction mechanism [38]. Our proposed mechanism begins with oxidation of the cyclic amines of diacetamide **27** to the Fagan amine-*N*-oxide orientation **26** (Scheme 18). Protonation in acidic media gives the imidols, upon loss of water. The double intramolecular nucleophilic imidol addition onto the iminum ion leads to the ring-fused imidazo[5,4-*f*]benzimidazole [27].

**Scheme 19:**
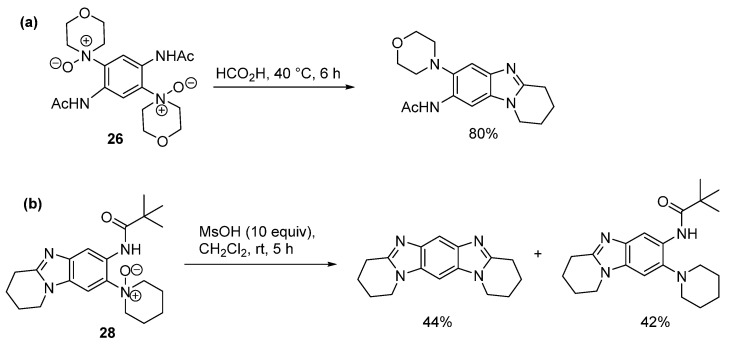
Acid-mediated cyclization of amine *N*-oxides to (**a**) benzimidazole [27] and (**b**) imidazo[4,5-*f*]benzimidazole [61].

An acid-catalyzed cyclization mechanism was proposed for conversion of dimorpholine *N*-oxide **26** to the [1,4]oxazino[4,3-*a*]benzimidazole (Scheme 19a) [27]. In the absence of an external oxidant, oxidation is possible through the internal conjugated system. MsOH allowed conversion of amine *N*-oxide **28** to the imidazo[4,5-*f*]benzimidazole, where presumably another molecule of **28** acts as oxidant (Scheme 19b) [61]. 

### 2.2. Reductions of Nitrobenzene-o-Cycloamines (Route B)

**Scheme 20:**
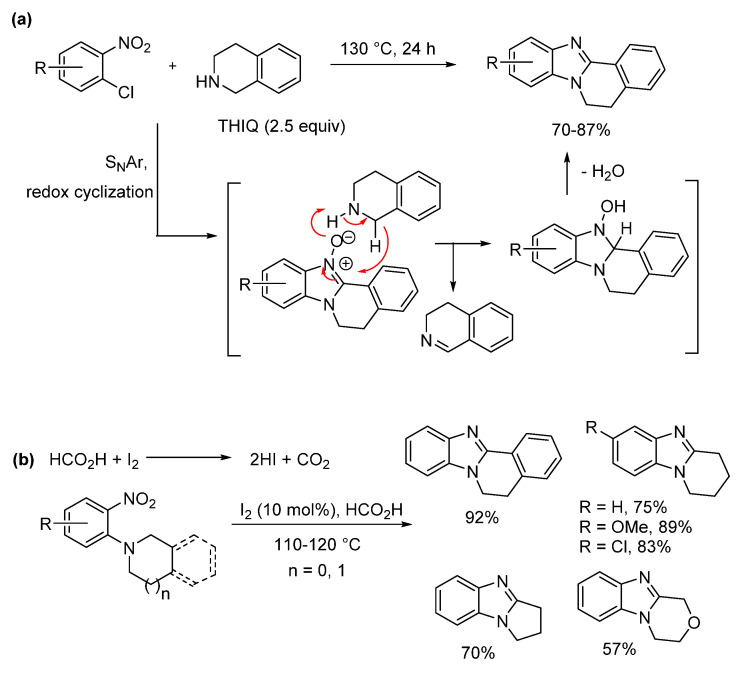
Reductive cyclizations using (**a**) thermal conditions [71] and (**b**) I_2_/HCO_2_H [72].

The reduction under acidic conditions of the aromatic nitro-group with cyclization onto the adjacent cycloamine substituent dates to the 1960s and employ ZnCl_2_/Ac_2_O [16,64,65], TiCl_3_/HCl [66], and Fe/AcOH [67]. There are cyclizations using Pd-catalysis with CO [68] or H_2_ [69]. Recent metal-free conditions use visible light, phenylthiourea as catalyst and PhSiH_3_ as reductant [70], and electrochemical cyclizations [52]. Thermal annulation using nitrobenzene substrates are possible with neat 1,2,3,4-tetrahydroisoquinoline (THIQ) (Scheme 20a) [71], and cyclizations occur using I_2_/HCO_2_H [72] (Scheme 20b). For the former reaction, the authors speculated on THIQ acting as a hydride donor after the initial S_N_Ar and redox cyclization, while HI generated in situ, is the active catalytic species in the latter reaction, acting as a strong Brønsted acid and reductant [71,72].

### 2.3. Using Aromatic Amidines, Lactams, and Isothiocyanates (Route C)

**Scheme 21:**
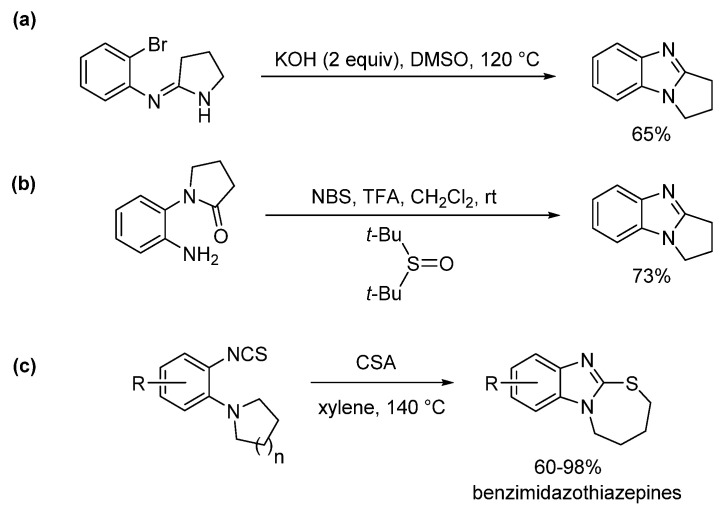
Synthesis from (**a**) *o*-haloarylamidine [75], (**b**) a lactam derivative of *o*-phenyldiamine [76], and (**c**) a rearrangement of aryl isothiocyanates [77].

Non-redox cyclizations of *o*-haloarylamidines give ring-fused benzimidazoles, using CuI as catalyst [73,74] (Scheme 21a) or strong base (Scheme 21b) [8,75], where nitrogen displaces the *o*-halogen. Pyrrolo[1,2-*a*]benzimidazole was reported from an *o*-lactam substituted aniline cyclization using di-*t*-butyl sulfoxide/NBS via an aza-Wittig type-reaction (Scheme 21b) [76]. 1-(2-Isothiocyanatophenyl)pyrrolidines cyclize under thermal acidic conditions to benzimidazothiazepines (Scheme 21c) [77].

**Scheme 22:**
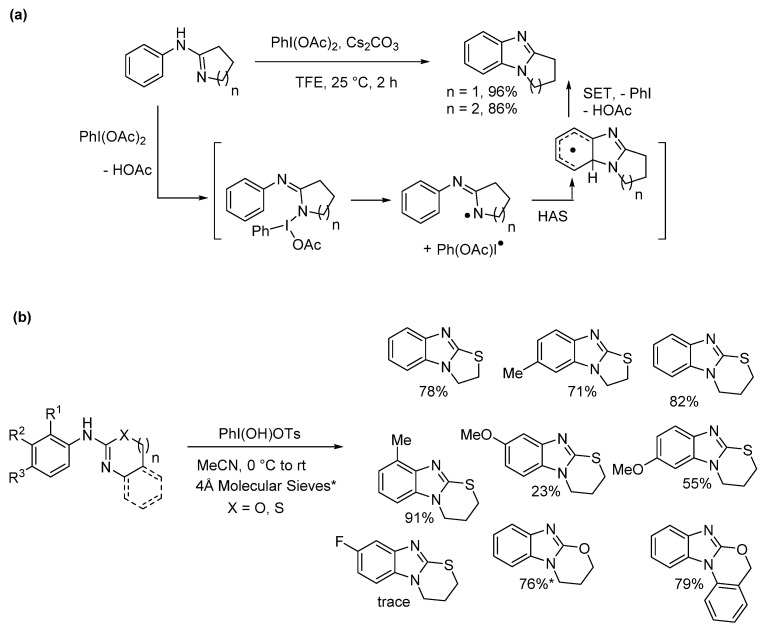
Cyclization of aryl amidines using (**a**) phenyliodine(III) diacetate [78] and (**b**) Kosher’s reagent [79].

Hypervalent iodine(III) reagents allow cyclization of aryl amidines onto non-functionalized benzenes (leaving group = H) [78,79]. The overall dehydrogenative process is proposed to proceed via a homolytic aromatic substitution (HAS) of nitrogen-centered radicals giving pyrrolo- and pyrido[1,2-*a*]benzimidazoles (Scheme 22a) [78]. Kosher’s reagent (PhI(OH)OTs) gave a range of thiazino and oxazino ring-fused benzimidazoles (Scheme 22b) [79]. The oxazino ring-fused benzimidazole required a longer reaction time, and the use of 4Å molecular sieves.

**Scheme 23:**
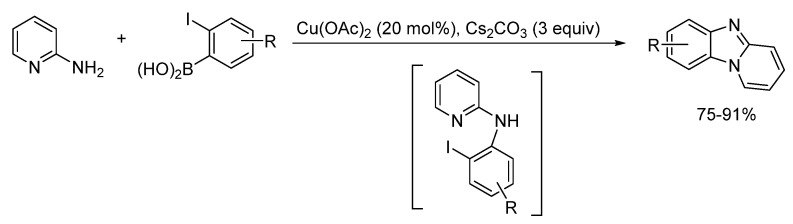
Synthesis of pyrido[1,2-*a*] benzimidazoles via amidine intermediates [80].

A combination of Chan−Lam and Ullmann type couplings were said to give the fully unsaturated pyrido[1,2-*a*]benzimidazoles using 2-aminopyridine and 2-iodoarylboronic acids via an amidine intermediate (Scheme 23) [80]. 

### 2.4. Condensations (Route D)

**Scheme 24:**
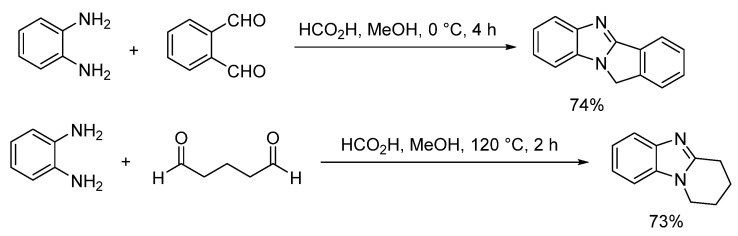
Acid-catalyzed condensation of 1,2-phenylenediamine with *o*-phthaldehyde and glutaraldehyde [82].

This includes the traditional bimolecular condensation of 1,2-phenylenediamines with aldehydes (Scheme 24) [81,82]. Rh-catalyzed cyclization of *N*-alkenyl-1,2-diaminobenzenes with CO/H_2_ gas gave alicyclic ring-fused benzimidazoles via a hydroformylation intermediate [83]. 

### 2.5. Annulations onto Benzimidazoles (Route E) 

A widely reported category for the synthesis of ring-fused benzimidazoles is annulations onto the 1- and 2-positions of benzimidazoles. This section reviews syntheses over the past 20 years, sub-divided according to the employed reaction (type) conditions.

#### 2.5.1. Base-Mediated Methods

**Scheme 25:**
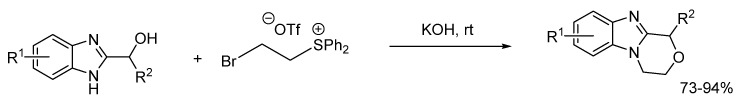
Annulations [4 + 2] of benzimidazoles using bromoethylsulfonium salt [86].

Early examples of annulation of the benzimidazole moiety cyclized 2-haloalkylbenzimidazoles under basic conditions through *N*-1 benzimidazole deprotonation to give pyrrolo-, pyrido- and azepino[1,2-*a*]benzimidazoles [84,85]. Bromoethylsulfonium salt disintegrated under basic conditions with loss of diphenyl sulfide, upon reaction with benzimidazole-2-methanols to give oxazino[4,3-*a*]benzimidazoles (Scheme 25) [86]. Extension of the latter sulfur ylide approach, achieved thiazino- and piperazino-adducts, but in lower yields.

#### 2.5.2. Transition Metal and Lewis Acid Catalyzed Methods

**Scheme 26:**
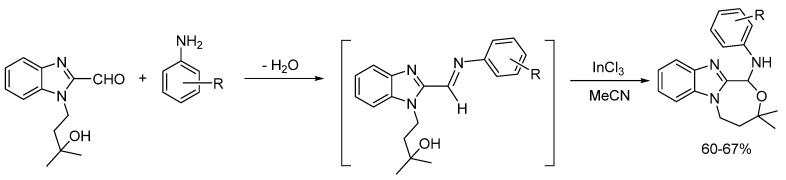
Synthesis of benzimidazole-fused 1,4-oxazepines [87].

InCl_3_-catalyzed the synthesis of benzimidazole-fused 1,4-oxazepines by intramolecular addition of a pendant alcohol onto an in situ-generated imine (Scheme 26) [87].

**Scheme 27:**
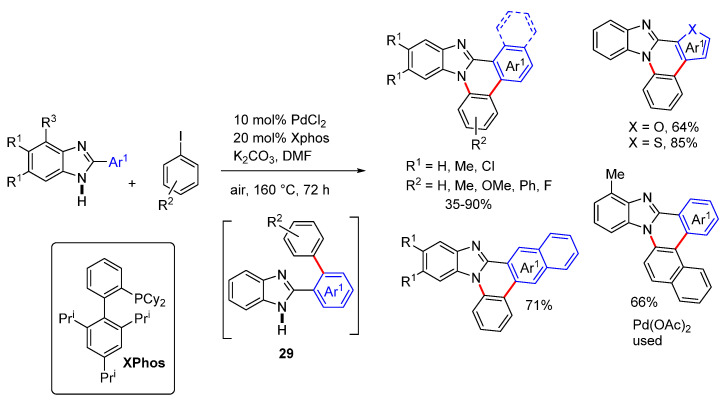
Pd(II)-catalyzed synthesis of benzimidazole-fused phenanthridines [88].

Starting from 2-arylbenzimidazoles and aryl iodides, a tandem Pd(II)-catalyzed C-H arylation and aerobic oxidative C-H amination sequence gave a variety of benzimidazole-fused phenanthridines (Scheme 27) [88]. Arylated intermediate **29** underwent C−H activation in a seven-membered palladacycle with the nitrogen of benzimidazole to yield the aminated products.

**Scheme 28:**
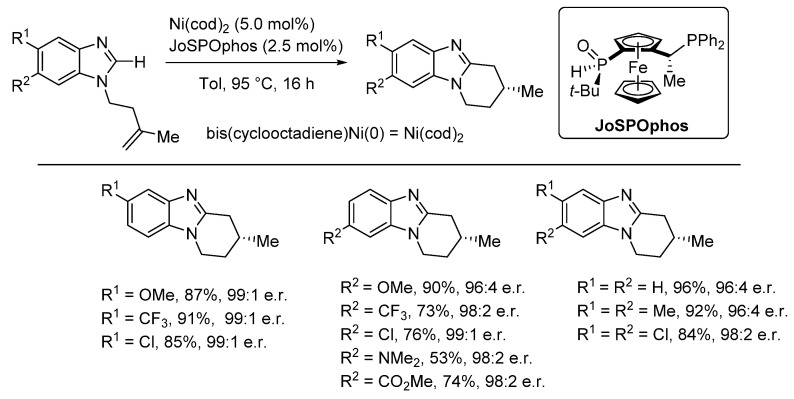
Asymmetric pyrido[1,2-*a*]benzimidazole syntheses [91].

There are several transition metal mediated benzimidazole-2-CH activated annulations including onto benzimidazole *N*-alkenyl [89,90,91] and *N*-alkynyl [92] substituents, with high entantioselectivity also achieved [90,91]. These include the use of chiral Ni/JoSPOphos manifolds (Scheme 28) [91]. 

**Scheme 29:**
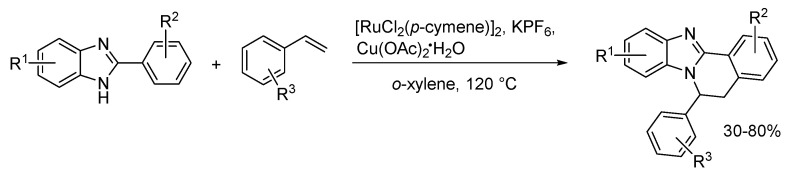
Annulations [4+2] of benzimidazoles using Ru(II)-NH and CH activation [93].

Ru(II)-catalyzed [4+2] annulations of 2-arylbenzimidazole with styrenes yielded benzimidazo[2,1-*a*]isoquinolines (Scheme 29) [93]. 

**Scheme 30:**
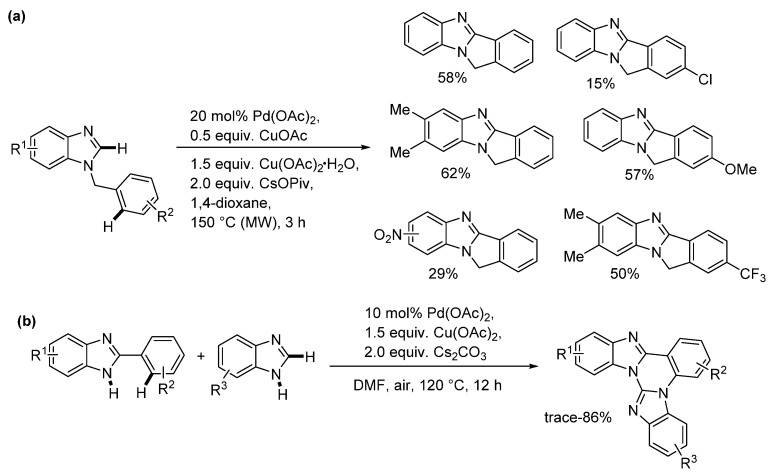
Pd-catalyzed dehydrogenative cross-couplings to give (**a**) 11*H*-isoindolo[2,1-*a*]benzimidazoles [94] and (**b**) benzimidazoquinazolines [95].

DeBoef et al. employed Pd(II)/Cu(I) catalysis with the oxidant of Cu(OAc)_2_ to effect oxidative dehydrogenative coupling of 1-benzyl substituted benzimidazoles (Scheme 30a) [94]. The Pd(II) C-H aryl-activation of 2-phenylbenzimidazoles with C-N coupling of two benzimidazole fragments gave benzimidazoquinazolines, with Cu(OAc)_2_ or air being the terminal oxidants (Scheme 30b) [95].

**Scheme 31:**
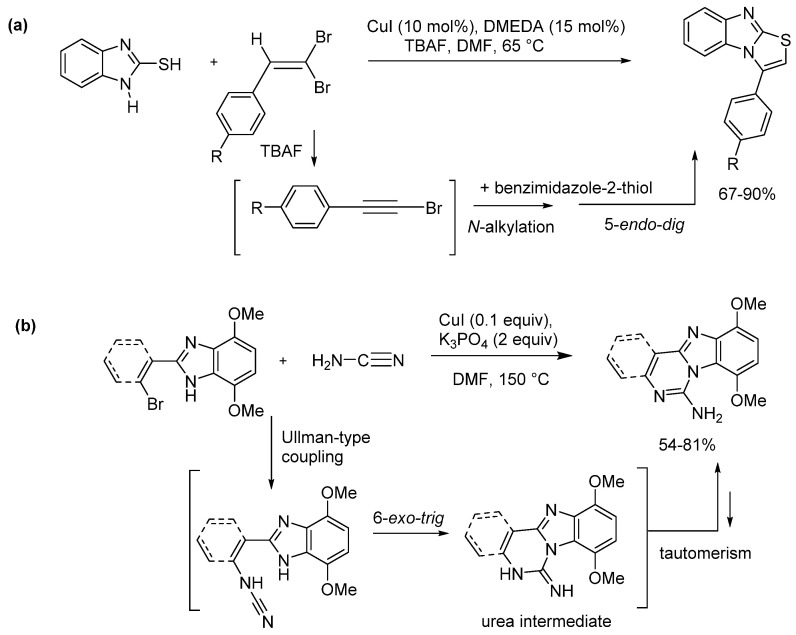
CuI-catalyzed annulations onto *N*-1 of benzimidazole using (**a**) 1,1-dibromoalkenes [96] and (**b**) cyanamide [97].

CuI-catalyzed an aminothiolation of 1,1-dibromoalkenes (Scheme 31a) [96]. The mechanism involves initial in situ dehydrobromination of the 1,1-dibromoalkene to 1-bromoalkyne. *N*-alkylation of the aromatic alkene and Cu-catalyzed C-S coupling or 5-*endo*-*dig* cyclization gives the annulated benzimidazole. More recently, 2-(2-bromoaryl)- and 2-(2-bromovinyl)benzimidazole substrates were coupled with cyanamide using CuI to give 2-aminoquinazoline and 2-aminopyrimidine hybrid structures (Scheme 31b) [97]. Similar conditions allowed CuI-mediated coupling and cyclization between 2-(2-bromoaryl)indoles and 2-aminoazoles [98], including 2-aminobenzimidazoles with loss of ammonia to give indole analogues of Scheme 30b adducts [95].

**Scheme 32:**
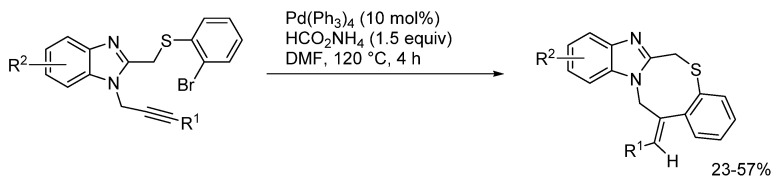
Pd-catalyzed macrocyclization to give benzimidazole-fused thiazocine [99].

A Pd-catalyzed reductive cyclization gave a variety of benzimidazole-fused thiazocine scaffolds (Scheme 32), but also thiazonines (9-membered rings), and thiazecines (10-membered) having an exocyclic double bond [99]. Ammonium formate is the hydrogen-donor.

#### 2.5.3. Radical Cyclization Methods

**Scheme 33:**
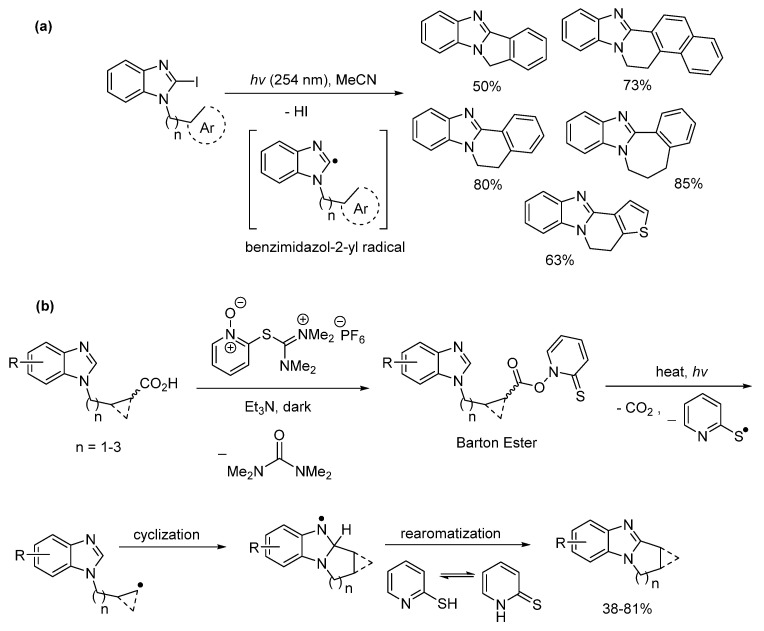
Radical initiator-free HAS using (**a**) UV-light [102] and (**b**) Barton ester intermediates [104].

We have reported annulations utilizing the benzimidazole-2-position to form nucleophilic ylide for condensation reactions [100,101] and the benzimidazol-2-yl radical for initiator-free photochemical HAS (Scheme 33a) [102]. Photochemical intramolecular HAS of the imidazol-2-yl radical are superior to analogous Bu_3_SnH-mediated reactions [103].

**Scheme 34:**
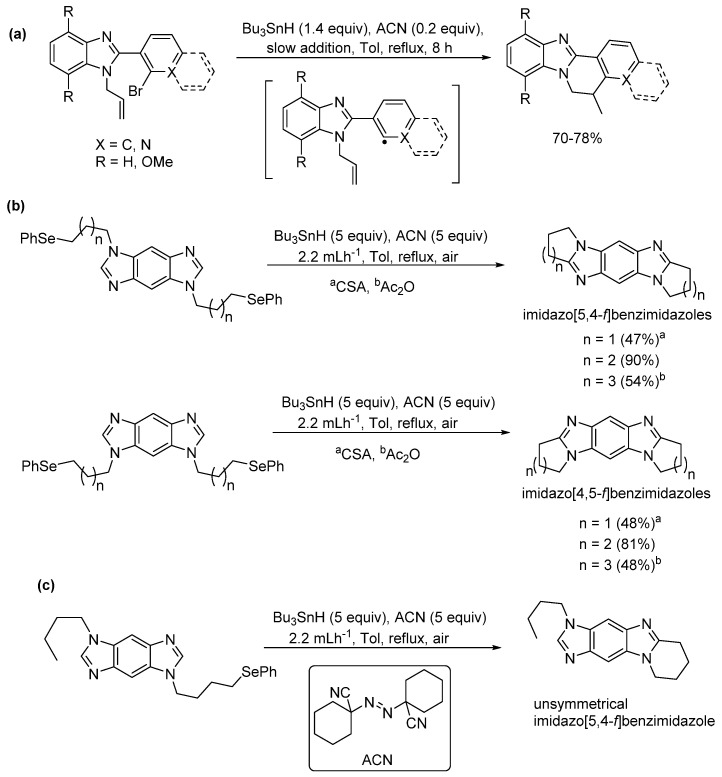
Bu_3_SnH-mediated formation of ring-fused benzimidazoles using (**a**) chain reaction [23,105], and non-chain reaction (**b**) double [26] and (**c**) single [28] HAS.

Bu_3_SnH-mediated chain reactions enabled 6-*exo*-*trig* cyclizations of aryl or pyridinyl radicals to yield a variety of aromatic-ring fused benzimidazoles [23,105] (Scheme 34a). HAS using alkyl radical cyclizations onto benzimidazole [21], imidazo[4,5-*f*]benzimidazole and imidazo[5,4-*f*]benzimidazole [26] proceed as non-chain reactions [106,107,108] requiring full equivalents of Bu_3_SnH and azo-initiator to give the aromatic products in moderate to high yields (Scheme 34b). Single ring-fused imidazo[5,4-*f*]benzimidazolequinones for anti-cancer evaluation studies, were also derived via this radical cyclization route (Scheme 34c) [28]. The latter cyclization required a substrate prepared by two separate alkylations of imidazobenzimidazole using 1-chlorobutane and 1-chloro-4-(phenylselenyl)butane. For Bu_3_SnH-mediated reactions, slow addition of radical initiators is required, which prevents Bu_3_SnH reduction of the cyclizing radical. Camphorsulfonic acid (CSA) or Ac_2_O are added to more difficult cyclizations, to activate the benzimidazole-2-position towards nucleophilic radical addition [21,26]. For the HAS onto imidazobenzimidazoles (Scheme 34b,c), exposing the reaction mixture to air for part of the reaction time gave higher yields, since oxygenated radicals are thought to be involved in the oxidative re-aromatization (or hydrogen atom-abstraction) step [26,108]. 

**Scheme 35:**
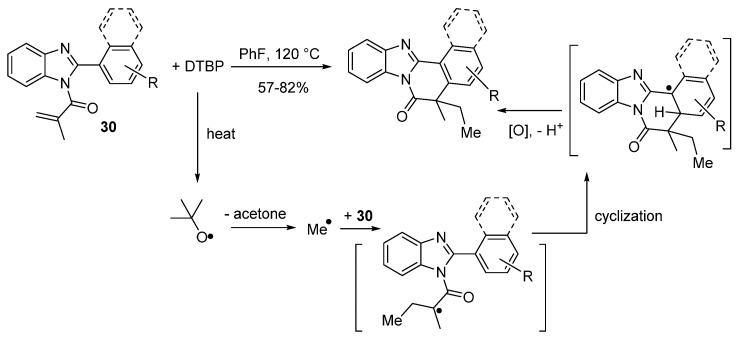
Di-*tert*-butyl peroxide (DTBP)-mediated oxidative HAS [110].

An alternative approach to HAS, is initiator-free, using in situ generated Barton ester intermediates derived from robust and readily accessible carboxylic acid substrates, giving five- to seven-membered alkyl and cyclopropyl radical cyclizations via a chain reaction mechanism (Scheme 33b) [104,109]. Most recently, benzimidazo[2,1-*a*]isoquinolin-6-ones were prepared by the addition of methyl radicals onto *N*-methacryloyl-2-phenylbenzimidazoles **30** using di-*tert*-butyl peroxide (DTBP) initiator (Scheme 35) [110]. The tertiary adduct radical undergoes six-membered cyclization followed by oxidation of the derived cyclized radical. Earlier, *tert*-butyl radicals generated from pivalic acid and several other carboxylic acids with K_2_S_2_O_8_/AgNO_3_ initiated an analogous cascade [111].

#### 2.5.4. Other Metal-Free Methods

**Scheme 36:**
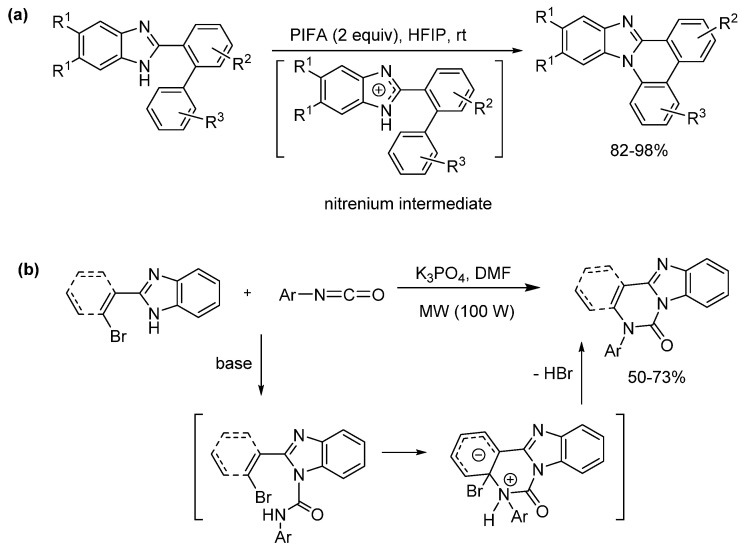
Annulations onto *N*-1 of benzimidazole using (**a**) PIFA [112] and (**b**) aryl isocyanates [113].

Mal et al. reported the use of PIFA (PhI(OCOCF_3_)_2_) for six-membered aromatic substitutions via a proposed nitrenium ion intermediate (Scheme 36a) [112]. Cho et al. made the reaction at *N*-1 of benzimidazole intermolecular, with addition onto aryl isocyanate and aromatic substitution of the carbamoyl-NH to give fused quinazolinones and pyrimidinones (Scheme 36b) [113]. 

**Scheme 37:**
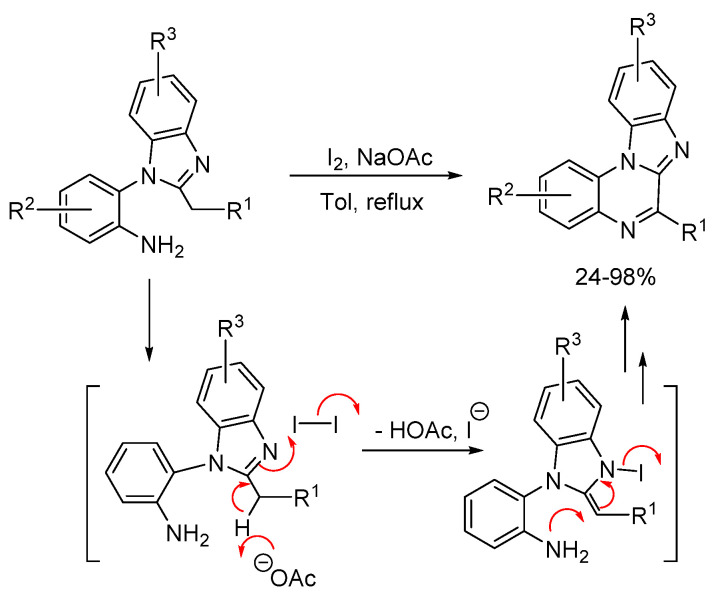
Condensation of 2-(benzimidazol-1-yl)anilines [114].

There are now several reports of benzimidazole syntheses using iodine in metal-free procedures [56,72,114]. Benz[4,5]imidazo[1,2-*a*]quinoxaline derivatives were obtained by an I_2_-mediated oxidative condensation of 2-(benzimidazol-1-yl)aniline substrates (Scheme 37) [114].

#### 2.5.5. Miscellaneous: Syntheses of Mitomycin Analogues 

**Scheme 38:**
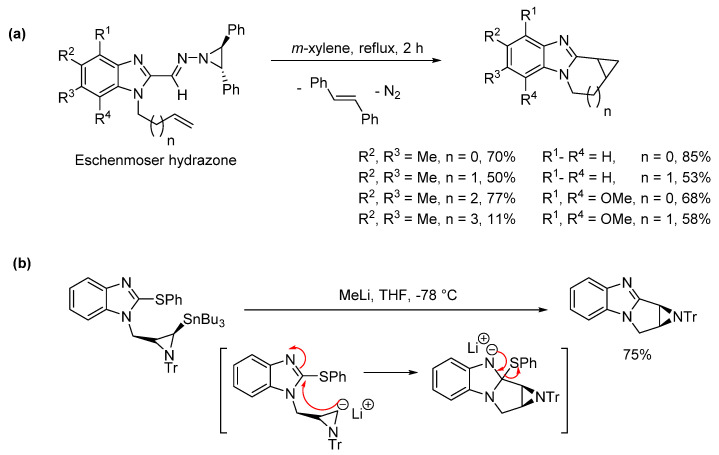
Synthesis of analogues of (**a**) cyclopropamitosenes [22,115,116] and (**b**) aziridinomitosene [14].

We reported the synthesis and anti-cancer evaluation of diazole analogues of MMC derivatives (Figure 3) and related indolequinones [14,21,22,115,116]. The formation of pyrrolo-, pyrido-, azepino- and azocino[1,2-*a*]benzimidazoles with a fused cyclopropane ring involves *N*-aziridinyl imines (Eschenmoser hydrazones) undergoing thermolysis with a loss of nitrogen and *trans*-stilbene (Scheme 38a) [22,115,116]. The formation of the aziridinomitosene analogue skeleton was via an intramolecular anionic aromatic *ipso*-substitution of an aziridinyl functionality onto the benzimidazol-2-yl position (Scheme 38b) [14]. Earlier the 2-phenylsulfanylbenzimidazole precursors gave five to seven-membered ring-fused benzimidazoles using alkyl radical cyclizations with substitution of the radical leaving group at the 2-position [117]. 

## 3. Conclusions

Over the past 20 years, significant advances in the synthesis of ring-fused benzimidazoles have occurred, notably using dehydrogenative coupling and radical cyclization. Transition metal catalysts achieve intramolecular and intermolecular aminations with benzimidazoles, with enantioselectivity for the former. Ring-fused benzimidazoles are prepared using hypervalent iodine(III) reagents and elemental iodine under metal-free conditions. There is an increasing use of sustainable and non-metal-mediated protocols, including photochemical, electrochemical, and thermal methods. Mild oxidative conditions tend to be more effective for preparing isoindoline and tetrahydroisoquinoline-fused scaffolds. There are effective methods for incorporating heteroatoms into the fused-ring, including N, O, and S atoms, and forming alicyclic rings with additional fused cyclopropane or delicate oxetane and aziridine rings. In terms of versatility, green chemistry, and value for money, it is difficult to beat the use of H_2_O_2_ in traditional oxidative cyclizations of *o*-(cycloamino)anilines. H_2_O_2_ in combination with HX generates the ordinarily inconvenient elemental halogen (X_2_) in situ, to mediate one-pot oxidative cyclization with halogenation. The latter includes one-pot approaches to potential antitumor ring-fused benzimidazolequinones from readily accessible anilines. Oxone is a cheap alternative, with technical and environmental benefits, including the circumvention of organic waste by-products. Our present work generates X_2_ in situ, by combining Oxone with benign NaX, to carry out one-pot halogenation with oxidative demethylation to give the dimeric quinones of ring-fused dimethoxybenzimidazole-benzimidazolequinones (DMBBQs) [118]. The DMBBQ scaffold offers unique regioselective functionalization opportunities for bis-quinone motifs, and these unique dimeric structures require investigation as bioreductive anti-tumor agents. 

Mechanisms for ring-fused benzimidazole and imidazobenzimidazole formation via the *t*-amino effect are now better defined. Recent studies have shown that acid and heat are unnecessary for oxidative cyclization via a nitrosobenzene intermediate using *o*-(cycloamino)anilines as substrates for benzimidazole formation. This offers opportunities for further investigations into the synthesis of ring-fused benzimidazoles under nonaggressive ambient conditions using commercial anilines as starting materials. While use of anilide derivatives results in a different mechanism via an amine-*N*-oxide intermediate. The anilide derivative and acidic conditions are mandatory for peroxide-mediated ring-fused imidazobenzimidazole preparations. To date ring-fused imidazobenzimidazoles have only been prepared via oxidative cyclizations of anilides and radical cyclizations onto imidazobenzimidazoles, surely new synthetic methods will merge for this interesting scaffold.

## Data Availability

Not applicable.
